# A novel rapid test for detecting antibody responses to *Loa loa* infections

**DOI:** 10.1371/journal.pntd.0005741

**Published:** 2017-07-27

**Authors:** Bijan Pedram, Valérie Pasquetto, Papa M. Drame, Yongchang Ji, Maria J. Gonzalez-Moa, Richard K. Baldwin, Thomas B. Nutman, Marco A. Biamonte

**Affiliations:** 1 Drugs & Diagnostics for Tropical Diseases, San Diego, California, United States of America; 2 Laboratory of Parasitic Diseases, National Institute of Allergy and Infectious Diseases, National Institutes of Health, Bethesda, Maryland, United States of America; 3 nanoComposix, San Diego, California, United States of America; Washington University School of Medicine, UNITED STATES

## Abstract

Ivermectin-based mass drug administration (MDA) programs have achieved remarkable success towards the elimination of onchocerciasis and lymphatic filariasis. However, their full implementation has been hindered in Central Africa by the occurrence of ivermectin-related severe adverse events (SAEs) in a subset of individuals with high circulating levels of *Loa loa* microfilariae. Extending MDA to areas with coincident *L*. *loa* infection is problematic, and inexpensive point-of-care tests for *L*. *loa* are acutely needed. Herein, we present a lateral flow assay (LFA) to identify subjects with a serological response to *Ll*-SXP-1, a specific and validated marker of *L*. *loa*. The test was evaluated on serum samples from patients infected with *L*. *loa* (n = 109) and other helminths (n = 204), as well as on uninfected controls (n = 77). When read with the naked eye, the test was 94% sensitive for *L*. *loa* infection and was 100% specific when sera from healthy endemic and non-endemic controls or from those with *S*. *stercoralis* infections were used as the comparators. When sera of patients with *O*. *volvulus*, *W*. *bancrofti*, or *M*. *perstans* were used as the comparators, the specificity of the LFA was 82%, 87%, and 88%, respectively. A companion smartphone reader allowed measurement of the test line intensities and establishment of cutoff values. With a cutoff of 600 Units, the assay sensitivity decreased to 71%, but the specificity increased to 96% for *O*. *volvulus*, 100% for *W*. *bancrofti*, and 100% for *M*. *perstans*-infected individuals. The LFA may find applications in refining the current maps of *L*. *loa* prevalence, which are needed to eliminate onchocerciasis and lymphatic filariasis from the African continent.

## Author summary

Loiasis affects over 10 million people in sub-Saharan Africa, and there are no commercial assays to detect *Loa loa* infection. New diagnostics for *L*. *loa* are urgently needed for two different purposes. First, although *L*. *loa* is generally a relatively asymptomatic infection, it has been associated with serious renal, cardiac, and neurological complications. Second, *L*. *loa* infection represents a major obstacle to the MDA-based elimination of river blindness and, to a lesser extent, of lymphatic filariasis. The programs to control and eliminate these parasites rely on mass administrations of ivermectin, a drug that has been associated with neurologic adverse events and sometimes death in patients with high levels of *L*. *loa* microfilariae. Herein, we present a novel lateral flow assay for *L*. *loa* infection. It is hoped that this test will help refine the current maps of loiasis, which will in turn allow optimization of programmatic decisions in the fight against *O*. *volvulus* and *W*. *bancrofti*, and ultimately against *L*. *loa* itself.

## Introduction

Loiasis, also known as African eye-worm disease, is a vector-borne parasitic infection caused by *Loa loa*, a filarial worm endemic to Central and West Africa [1]. Epidemiological data collected from 11 African countries indicates that at least 10 million people are infected [2]. In certain communities, the prevalence of loiasis is astounding, with over 40% of the population displaying microfilariae [3].

*L*. *loa* is transmitted by daytime biting flies of the genus *Chrysops* during a blood meal. The fly injects into the human host infective larvae (L3 development stage) that develop over time into adult worms. These then mate to produce microfilariae (mf) that circulate in peripheral blood [1,4]. The major clinical manifestations of loiasis are Calabar swellings (evanescent episodic angioedema) and the subconjunctival migration of the adult worm (eye-worm). Less specific manifestations include urticaria, pruritus, myalgias, and arthralgia [5]. Moreover, *L*. *loa* infection can cause renal, cardiac, pulmonary and neurological diseases [6] and a recent study found *L*. *loa* infection to be associated with a decreased life expectancy [7]. Despite this, loiasis is still considered a benign disease and does not appear on the World Health Organization’s official list of Neglected Tropical Diseases [6].

Loiasis is a major public health issue because of its geographic overlap with onchocerciasis and lymphatic filariasis [8]. The international community has deployed intense efforts to eliminate these two diseases through vector control and mass drug administration (MDA) programs. Over 600 million doses of ivermectin (Mectizan) are distributed annually, as monotherapy against onchocerciasis, and in combination with albendazole or diethylcarbamazine against lymphatic filariasis [9]. Yet, ivermectin can lead to serious and occasionally fatal adverse neurological reactions in people infected with *L*. *loa*. The severe adverse events (SAEs) occur when the *L*. *loa* microfilaremia exceeds 20,000–30,000 mf/mL. These SAEs are thought to result from mf dying within the vessels of the central nervous system and from the ensuing eosinophil-rich inflammatory response, a process that can result in encephalopathy [10,11]. The initial signs of encephalopathy –confusion, agitation, lethargy, dysarthria, mutism, and urinary incontinence– appear 2 to 3 days post-dosage and can progress into coma and, eventually, death [12]. While in *L*. *loa* co-endemic areas, bancroftian filariasis can be addressed with albendazole alone [9], there are no chemotherapeutic agents other than ivermectin available for MDA against onchocerciasis [13]. Thus, loiasis poses serious ethical and logistic difficulties to onchocerciasis elimination programs. The current guidelines of the Mectizan Expert Committee [12] allow ivermectin-based MDA to proceed in regions of *Loa* co-incidence, but only if the prevalence of onchocerciasis exceeds 40% based on the presence of *O*. *volvulus* microfilariae in skin snips, or 20% based on the presence of palpable nodules in adult males. Even so, a system of pharmacovigilance must be in place to intercept post-ivermectin SAEs. Otherwise, a more demanding test-and-(not)-treat intervention strategy must be implemented, where every person in the community must be tested for *Loa* microfilaremia to determine if he or she can receive ivermectin.

Point-of-care tests for loiasis are needed to define more precisely which individuals and which communities are eligible to receive ivermectin. While broad *L*. *loa* prevalence maps exist [2], more granular maps may help decide which communities can be safely included in MDA programs and which ones should be approached with a more labor-intensive test-and-(not)-treat approach. Substantial efforts of data collection, statistical analysis, and modelling are currently underway to examine if one can define a limit of *Loa* prevalence under which ivermectin MDA would be safe and ethical [14].

Definitive diagnosis of loiasis can be done by the identification of the adult worm in the eye or after its removal from under the skin or by morphological identification of the mf in blood smears, but these are low throughput methods inadequate for mapping purposes. Molecular methods such as loop-mediated isothermal amplification (LAMP) [15,16] and quantitative PCR (qPCR) [17] assays are credible alternatives to microscopy-based techniques since they combine a high degree of sensitivity and specificity with high throughput capabilities. However, molecular methods remain impractical for rapid testing at the point-of-care and are relatively expensive. Proteomic and immunological analyses of *L*. *loa* infected human samples have identified *L*. *loa* specific biomarkers. Of these, the *L*. *loa* protein LOAG_16297 has been introduced as a promising biomarker for future antigen-based tests, but it has not yet been applied for point-of-care testing [18]. Finally, a portable smartphone-based microscope (LoaScope) has been developed to simplify and accelerate the counting of *L*. *loa* microfilariae at the point-of-care [19].

Additionally, there are well-established immunoassays [20] [21] that detect circulating antibodies specific for the *Ll*-SXP-1 gene product. *Ll*-SXP-1 (GenBank accession number: EFO21235.1) is a 168 amino acid protein of unknown function expressed by all stages of the *L*. *loa* parasite. Orthologs of the *Ll*-SXP-1 gene have been identified in the genome of *O*. *volvulus* (Ov17), *W*. *bancrofti* (Wb-SXP-1) [22–25], and *B*. *malayi* (Bm14, also known as BmM14) [26,27], which display a 51–53% sequence identity to *Ll*-SXP-1 (S1 Table). The *M*. *perstans* genome has not been sequenced yet. An ELISA assay configured to detect total IgG to *Ll*-SXP-1 was 67% sensitive and 81% specific when patients infected with other filarial diseases were used as comparators [20]. When the test was restricted to detecting only the IgG4 isotype, the sensitivity decreased to 47% but the specificity against other filariae increased to 99% [20]. Similarly, a different type of immunoassay format (LIPS) was configured to detect either total IgG or IgG4. These LIPS assays dramatically increased the sensitivity (93–100%) but at a loss of specificity (78–81%), even in the IgG4 variant [21] (S2 Table). Herein, we adapt the validated *Ll*-SXP-1 methodology to a simple and inexpensive LFA to assess exposure to *L*. *loa*.

## Methods

### Ethics statement

The article reports test results on human sera. All samples were collected from subjects as part of registered protocols approved by the Institutional Review Boards of the National Institute of Allergy and Infectious Diseases, National Institutes of Health, collected under NCT00001230, NCT00342576, or 92-I-0155 (inactive). Some samples were collected as part of a large international field project approved by their respective governments. Written informed consent was obtained from all subjects.

### Technology and anatomy of the Loa Antibody Rapid Test

The Loa Antibody Rapid Test contains a test strip within a plastic cassette, which has a window and a single sample port. The test strip consists of four abutting components: a blood-filter (Ahlstrom, Alpharetta, GA), where the sample is deposited, and which prevents erythrocytes from migrating to the rest of the test strip, a fiberglass pad (Ahlstrom) in which reporter nanoparticles are dried together with other reagents in a sugar matrix, a nitrocellulose strip containing the test and control lines (EMD Millipore, Billerica, MA), and an absorbent pad to wick excess moisture (Ahlstrom).

The Loa Antibody Rapid Test features novel reporter nanoparticles consisting of a 20 nm thick gold shell encapsulating a spherical 110 nm silica core (nanoComposix, San Diego, CA). The optical properties of the particles can be modulated by varying the shell thickness. The nanoshells employed in the Loa Antibody Rapid Test are blue-to-black and have a per-particle extinction coefficient 35 times higher than that of red 40 nm gold commonly employed in other commercial LFAs. Consequently, they appear darker to the human eye even when 35 times more diluted. This translates into an increase in analytical sensitivity of 2–10 fold, depending on the exact assay. The low-density silica interior decreases the overall density of the particles so that they are easily re-suspended in water and can flow unimpeded through the LFA membrane. The Loa Antibody Rapid Test is, to our knowledge, the first LFA to incorporate gold nanoshells.

The Loa Antibody Rapid Test has a “bridged” design, with recombinant *Ll*-SXP-1 localized both at the surface of the gold nanoshells and at the test line. To be consistent with previous work [20], a 148-amino acid partial sequence of *Ll-*SXP-1 was used (GenBank accession no: AAG09181.1). The design leverages the bivalency of antibodies. When an IgG antibody specific for *Ll*-SXP-1 is present, one of its Fab portions can bind the reporter nanoparticle while the other can be captured by the test line, thus leading to an accumulation of nanoparticles at the test line, and therefore to a visual signal. This design can, at least in theory, detect any antibody irrespectively of its isotype. For procedural control purposes, the Loa Antibody Rapid Test also contains nanoparticles conjugated to an anti-fluorescein antibody and a control line of fluorescein-BSA. Thus, if the eluent is applied properly, a control line will always be formed, indicating that the test has developed properly.

### General procedure

The assay is run by laying the device flat (*e*.*g*. on a table). Next, 5 μL of serum, plasma, or whole blood are placed in the port by means of a calibrated micropipette, followed by 2 drops of eluent (52 μL). The assay is a single-port design and sample and eluent are added to the same port. The assay is read after 20 minutes and readout remains valid for up to 1 hour, provided that the test strip remains wet during that time. If the assay is deliberately left to dry overnight, it should be re-wetted with 1 drop of eluent (26 μL) before being read. In experiments where the *Loa* infected sample was diluted with negative serum, delipidized serum from uninfected North American individuals was utilized (ConeBio, Seguin, TX). All testing was performed at ambient temperature and humidity (22°C, 40–55% relative humidity).

### Reader

The intensities of the test and control lines were quantified with a HRDR-200 smartphone-based chromatographic test reader (Cellmic, Los Angeles, CA). The reader was programmed using Cellmic’s Test Developer Software (TDS) V3.7.0_160420 NK. The raw output values were obtained by integrating the Area Under the Peak (AUP) of zone intensities within a 10%-width security margin around each zone. The raw values are expressed as reader units (RU) derived from the calculated AUP. Test results were exported from the reader to Microsoft Excel using Cellmic’s Test Explorer Software (TES). Each test strip was visually validated for potential physical shifts in TES and further numerically analyzed in Microsoft Excel.

### Patient populations

Well-characterized pools of sera were used to optimize the LFA and to test reproducibility. The final device was tested in a blinded fashion using individual serum samples from patients with documented patent *L*. *loa* infection from Cameroon, Gabon, Benin, and the Central African Republic (*n* = 109), *W*. *bancrofti* infection from India and the Cook Islands (*n* = 49), *O*. *volvulus* infection from Ecuador, Guatemala and Ghana (*n* = 99), *M*. *perstans* infection from an onchocerciasis-negative area of Mali (n = 16), and *S*. *stercoralis* from South America and Southeast Asia (*n* = 40). Additional control sera were used from healthy controls from *Loa*-non-endemic regions of Africa (Mali, Ghana n = 28) and from North America who had never traveled outside North America (*n* = 49). The parasitological diagnosis of all filarial infections was made based on the demonstration of mf in the blood (for *L*. *loa*, *W*. *bancrofti*, and *M*. *perstans*), in the skin (for *O*. *volvulus*), or the detection of larvae in stool samples (*S*. *stercoralis*). For the few amicrofilaremic individuals with *L*. *loa*, a definitive diagnosis was made based on standardized criteria: presence of an eyeworm or Calabar swelling in individuals with a relevant exposure history [5].

### Statistical methods

All data were analyzed using GraphPad Prism (version 7.0) software. Groups of variables were analyzed for statistically significant differences using the Mann-Whitney U test. Correlations between variables were analyzed using the non-parametric 1-tail Spearman rank correlation test. Sensitivity and specificity values were calculated using Fisher’s exact test, and their 95% confidence intervals were determined by the Brown-Wilson method.

## Results

### Analytical sensitivity

**Fig 1:**
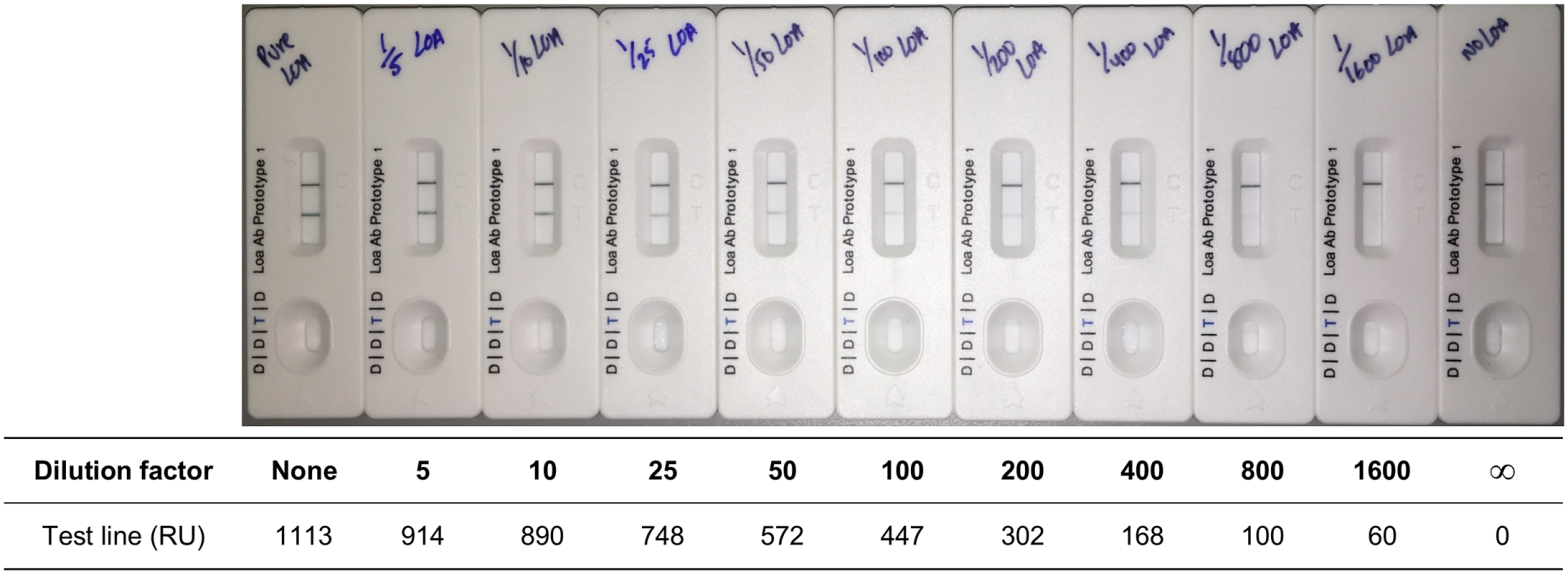
Analytical sensitivity of the Loa Antibody Rapid Test. The test was run as per the General Procedure, with 5 μL of undiluted pooled *L*. *loa* sera, (left), 5 μL pooled *L*. *loa* sera serially diluted in negative serum from uninfected North American individuals, with dilution factors of up to 1:1600 (center), or 5 μL pure negative delipidized serum (right). The test lines were quantified with the smartphone reader. The data is reported in reader units (RUs).

The analytical sensitivity of the Loa Antibody Rapid Test was assessed as per the General Procedure with pooled *L*. *loa* sera, either undiluted or serially diluted with negative serum from uninfected North American individuals. The signal was measured after 20 minutes with the Cellmic smartphone reader. When tested with undiluted pooled *L*. *loa* sera, the assay gave a sharp test line (1113 RUs), comparable in visual intensity to the control line (Fig 1). The signal remained sharp after a 5-fold and 10-fold dilution (914 and 890 RUs, respectively). Upon further dilutions of the test sample, the test line intensity gradually decreased. At a 100-fold dilution, the signal was clearly visible but weaker (447 RUs). The limit of eye detection was reached at an ~ 800-fold dilution (100 RUs).

### Time to readout

**Fig 2:**
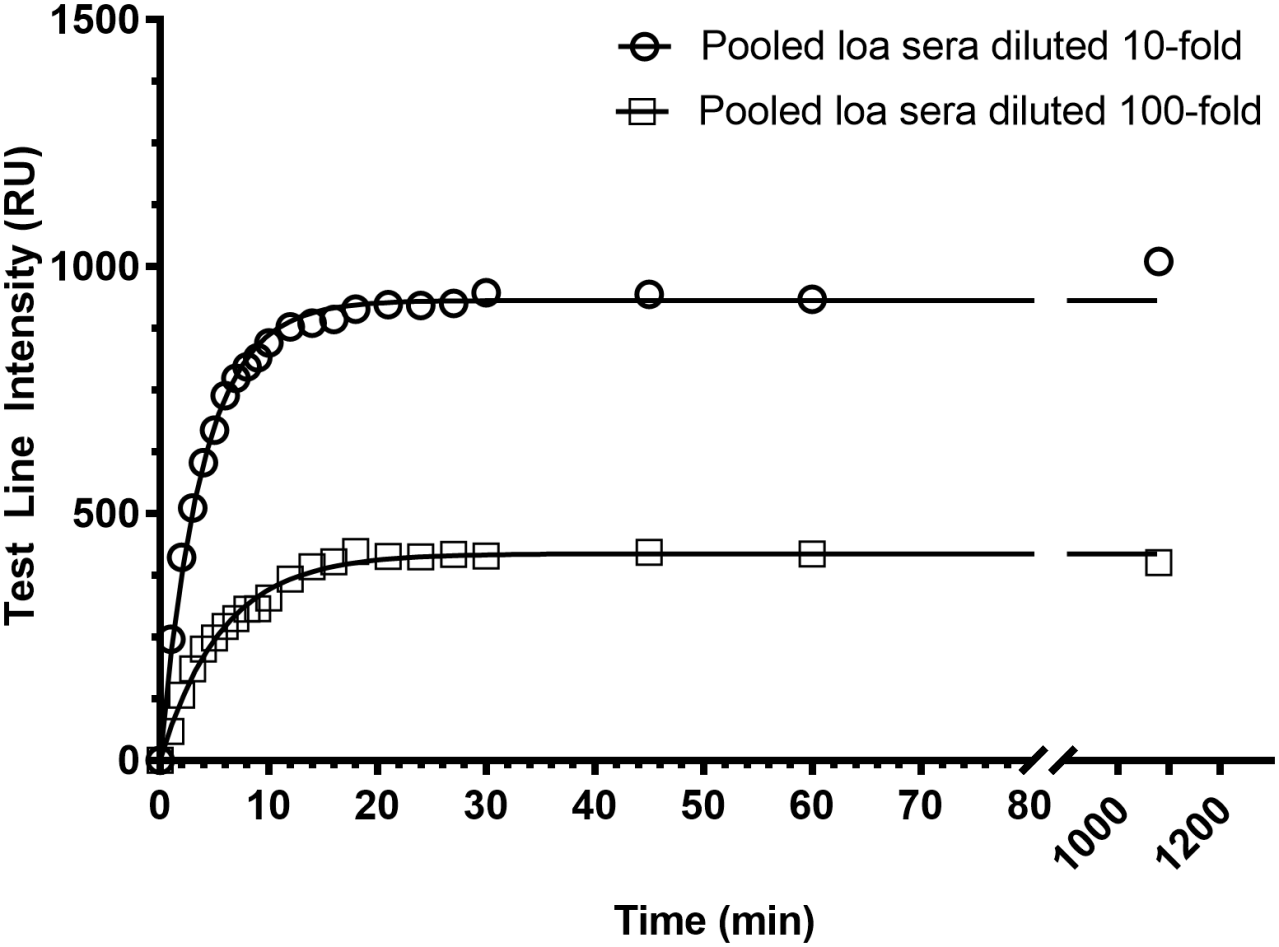
Test Line intensity as a function of time. The Loa Antibody Rapid Test was run as per the General Procedure, with 5 μL of pooled *L*. *loa* sera, diluted either 10- or 100-fold in negative human serum. The test line was quantified at different time points, with T = 0 being defined as the moment when the eluent was added.

The pool of *L*. *loa* sera was tested at two different dilutions (10- and 100-fold). Five microliters were deposited on the assay, followed by 2 drops (52 μL) of eluent. The test line developed rapidly and was clearly visible within 2 minutes. The signal increased over time, was half-developed after 5 minutes, and reached a plateau after 20 minutes (Fig 2).

### Reader and test strip reproducibility

A single device was run with the pool of *L*. *loa* sera (5 μL) for 30 min, and the same test line was measured 25 consecutive times with the reader. The mean value and standard deviation of the 25 reads were 1110 ±7 RUs, corresponding to a coefficient of variation CV = 0.7% (Table 1). The experiment was repeated using 5 μL of the same pooled *L*. *loa* serum sample, diluted either 10-fold or 100-fold with negative human serum. The mean signal decreased, while the standard deviation remained at 8–10 RUs, leading to CV values of 1.2–1.7%. It can be concluded that the reader gives highly reproducible readouts.

**Table 1:** Reproducibility of the smartphone reader. Pooled *L*. *loa* sera were tested at different dilutions on a given cassette, and the test line intensity was measured 25 consecutive times. The table reports the mean value and standard deviation in reader units, and coefficient of variation in %. Calculating a CV on negative serum is not meaningful since the denominator is close to zero.

Dilution factor	undiluted	10	100	Negative sample
Mean ± SD (n = 25)	1110 ± 7	852 ± 10	475 ± 8	0.8 ± 1.7
CV (%)	0.7	1.2	1.7	n/a

Having established that the reader gives highly reproducible results, we next evaluated the reproducibility of cassettes coming from a single manufacturing lot. Ten cassettes were randomly selected and tested with 5 μL of pooled *L*. *loa* sera, either undiluted, diluted 10-fold, or diluted 100-fold with negative serum prior to application. The standard deviations were in the range of 69–76 RUs, corresponding to CV values in the 6–19% range (Table 2). Furthermore, the control lines were tightly grouped with a median value of 1321 RUs (S1 Fig).

**Table 2:** Reproducibility of the test strips. Pooled *L*. *loa* sera were tested at different dilutions, each time on 10 different cassettes randomly selected from the same manufacturing lot. The test lines were read after 20–30 minutes with the smartphone reader.

Dilution factor	undiluted	10	100	Negative Sample
Mean ± SD (n = 10)	1110 ± 69	1010 ± 70	401 ± 76	0 ± 0
CV (%)	6.2	6.9	19	n/a

### Quantification of specific anti-*L*. *loa* antibody levels in clinical samples

The Loa Antibody Rapid Test was evaluated at the National Institutes of Health in the Laboratory of Parasitic Diseases using a set of cryopreserved human sera. For logistic reasons, the tests were let dry at the end of the run and were rewetted before scoring. The drying/rewetting process decreases the test line intensity by up to 20% but still gives reproducible and interpretable results.

**Fig 3:**
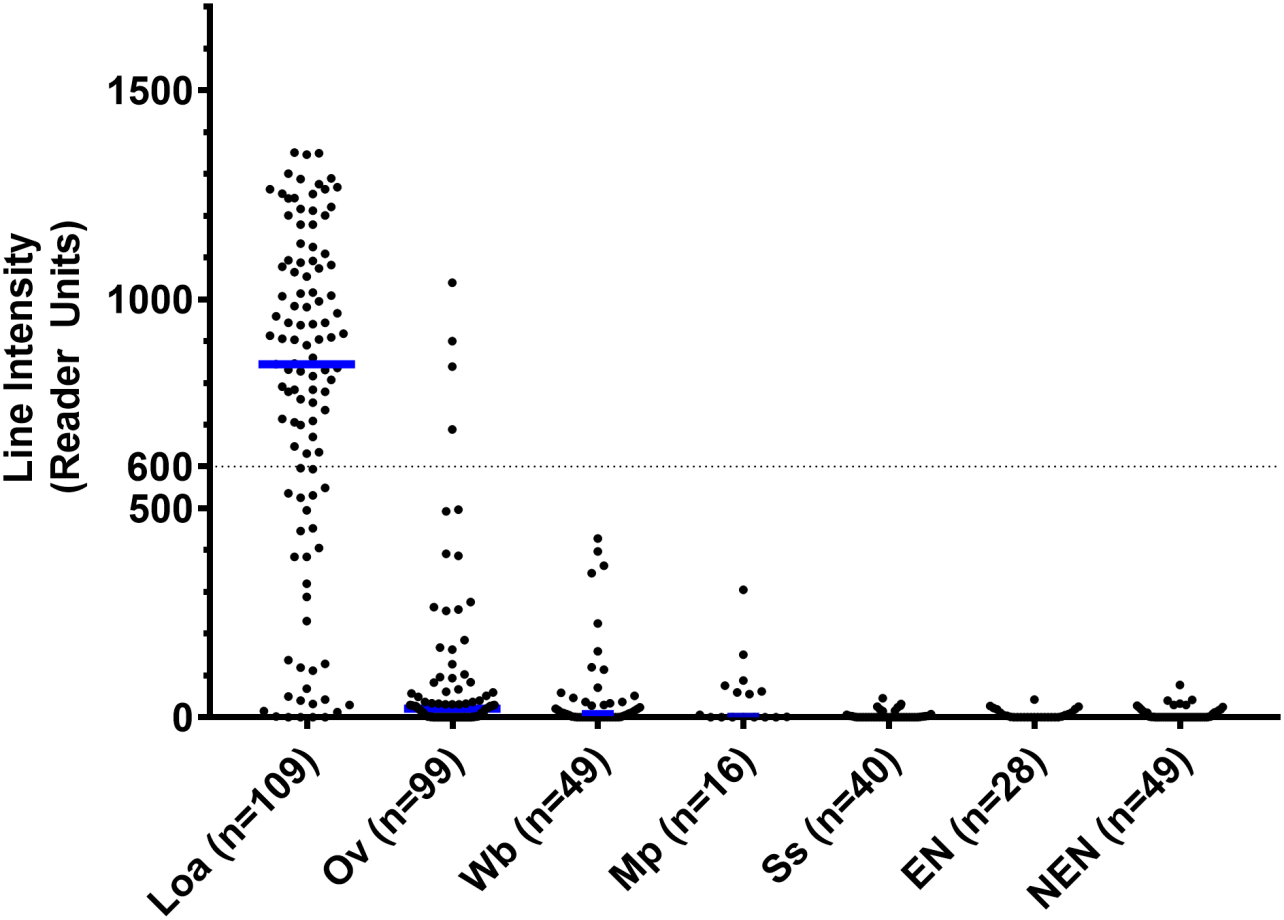
Control and test line intensities by disease. The scatter plot shows individual data points, with median values as blue horizontal bars. The median value for the *L*. *loa* samples was 845. A possible cutoff value of 600 RUs is shown as a dotted line. Ov = *O*. *volvulus*, Wb = *W*. *bancrofti*, Mp = *M*. *perstans*, Ss = *S*. *stercoralis*, EN = Endemic Normals, NEN = Non-Endemic Normals.

Fig 3 summarizes the clinical data. Most samples of sera from those with *L*. *loa* infection gave sharp signals, with a median value of 845 RUs. Onchocerciasis samples cross-reacted to a small degree with *Ll*-SXP-1 but there was a clear differentiation in the RUs between the two groups (P value < 0.0001 in a Mann-Whitney U Test). The onchocerciasis group gave substantially weaker signals and a median value of 21 RUs—either not detectable or barely detectable with the naked eye. Similarly, the bancroftian filariasis samples partially cross-reacted with the *L*. *loa* samples, but were even weaker with a median value of 10 RUs (undetectable by the naked eye, P < 0.0001). *M*. *perstans* samples gave even weaker signals, with a median value of 3.5 RU (P < 0.0001). *Strongyloides* samples as well as endemic and non-endemic normal controls were all negative by eye (P < 0.0001).

### Sensitivity and specificity of the LFA using clinical serum samples

**Fig 4:**
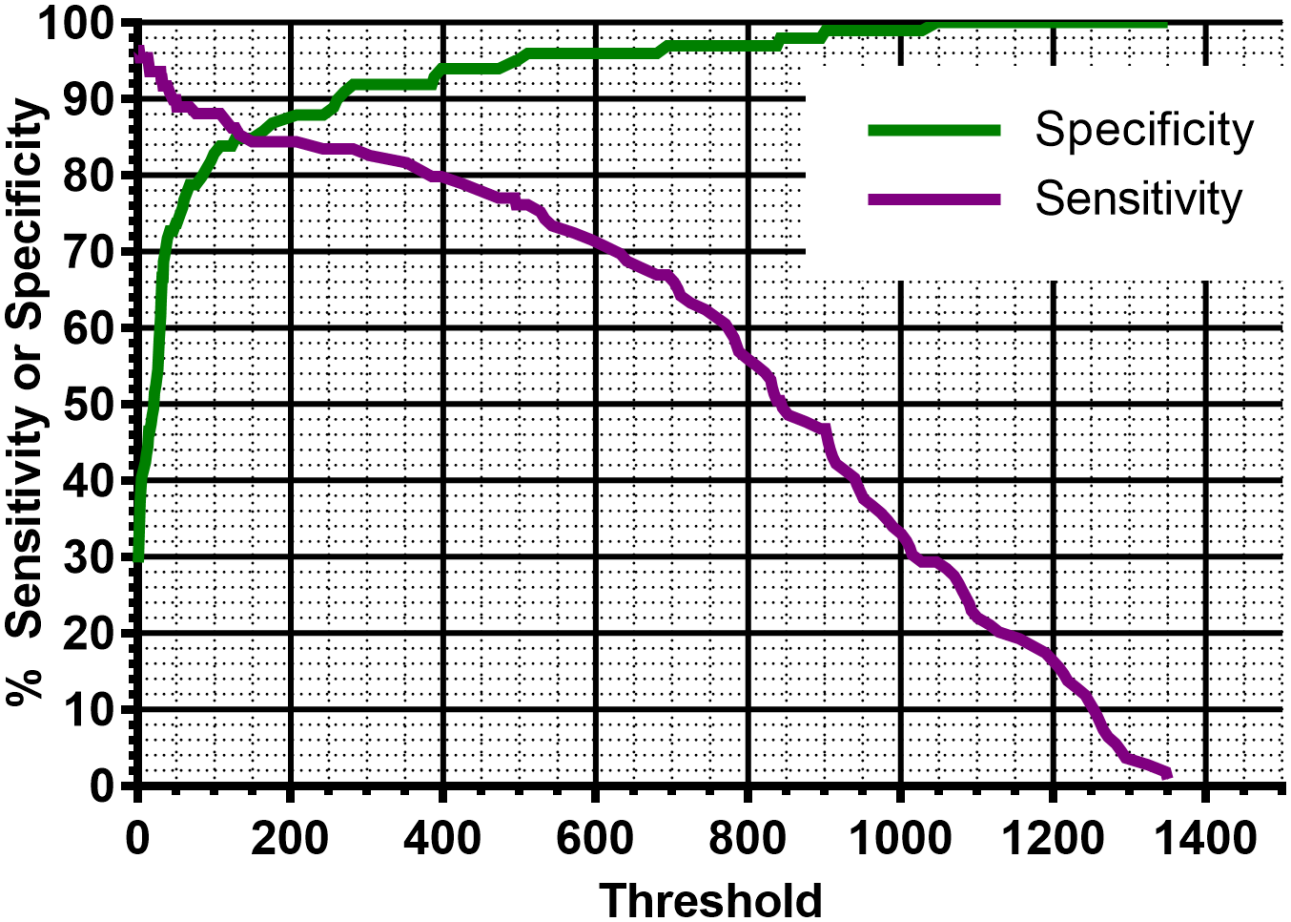
Sensitivity and specificity versus *O*. *volvulus* as a function of the cutoff value.

When read with the naked eye, the test was 94% sensitive for *L*. *loa*. The specificity was 82% *vs*. *O*. *volvulus*, 84% *vs*. *W*. *bancroft*i, 88% *vs*. *M*. *perstans*, and 100% *vs*. *S*. *stercoralis*, endemic normal, and non-endemic normal samples (Table 3). The LFA reader offers the remarkable advantage of establishing an objective cutoff value. By adjusting the cutoff value, the assay results can be tuned to modulate the sensitivity and specificity to the needs of the end-users (Table 3). A cutoff of 100 RUs is just above what can be detected by the unaided eye and provides results similar to the visual scoring: 88% sensitivity, 100% specificity when compared to normal, uninfected sera, and 83–88% specificity towards *W*. *bancrofti*, and *M*. *perstans*. With a cutoff of 200 RUs, the sensitivity is 84% and the specificity towards the other filariae is in the 89–94% range. By increasing the cutoff to 600 RUs, the sensitivity decreases to 71%, but the specificity towards other filarial species increases to 96–100%. Receiver Operating Characteristic (ROC) curves (Fig 4 and S2 Fig) allow to examine in more detail the effect of the cutoff on the sensitivity and specificity.

**Table 3:** Sensitivity and specificity at different cutoff values. The cutoff values are expressed in Reader Units (RUs). Sensitivity and specificity values are reported in %, with the 95% confidence intervals are reported in brackets. Ov = *O*. *volvulus*, Wb = *W*. *bancrofti*, Mp = *M*. *perstans*, Ss = *S*. *stercoralis*, EN = Endemic Normals, NEN = Non-Endemic Normals.

Cutoff	Visual	100 RUs	200 RUs	400 RUs	600 RUs
Sensitivity Loa (%)	94 (88–97)	88 (81–93)	84 (76–90)	80 (71–86)	71 (62–78)
Specificity Ov (%)	82 (73–88)	83 (74–89)	89 (10–94)	94 (87–97)	96 (90–98)
Specificity Wb (%)	87 (75–94)	84 (71–92)	90 (78–96)	98 (89–100)	100 (93–100)
Specificity Mp (%)	88 (64–98)	88 (64–98)	94 (72–100)	100 (91–100)	100 (91–100)
Specificity Ss (%)	100 (91–100)	100 (91–100)	100 (91–100)	100 (91–100)	100 (91–100)
Specificity EN and NEN (%)	100 (95–100)	100 (95–100)	100 (95–100)	100 (95–100)	100 (95–100)

### Relationship between antibody LFA response and microfilaremia

**Fig 5:**
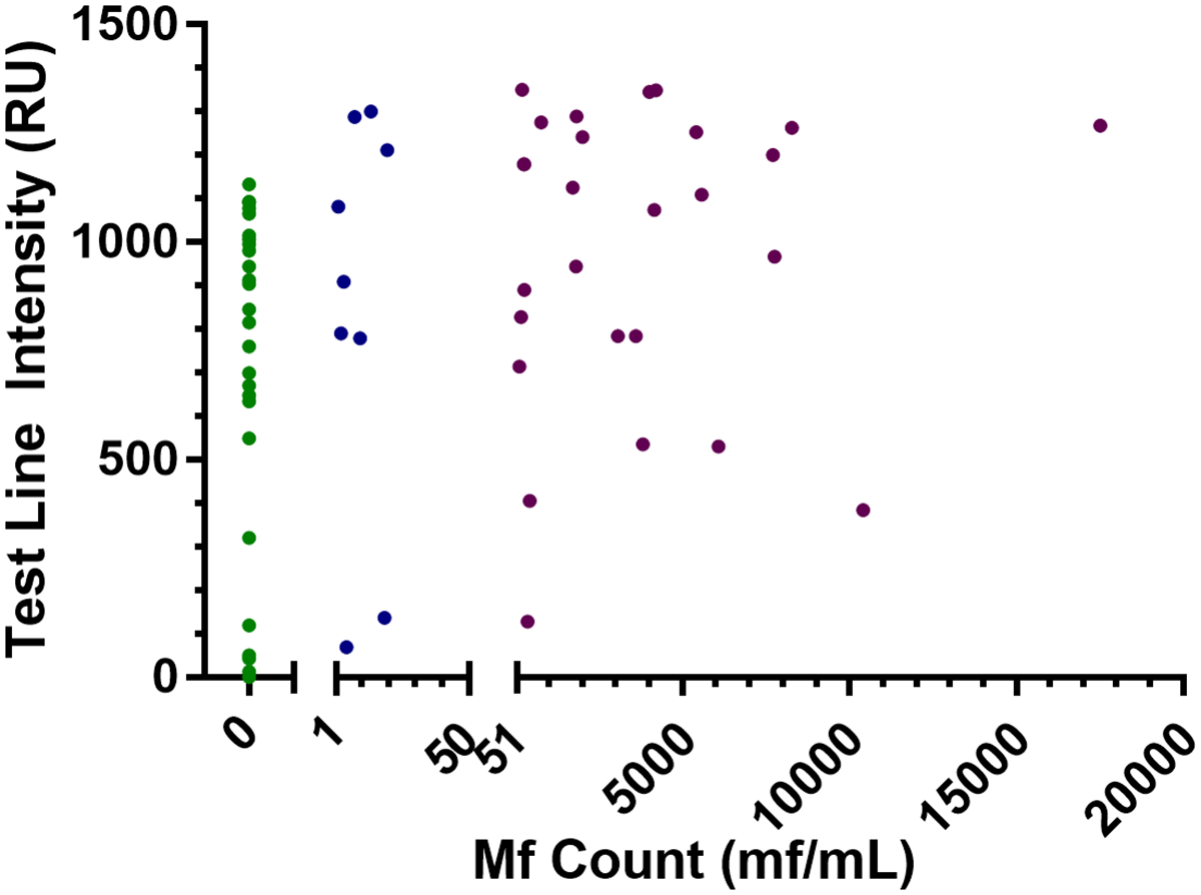
Relationship between test line intensity and microfilaremia. The scatter plot reports the intensity of the test line as a function of mf/mL count in the blood of 62 patients. The data set includes 26 includes amicrofilaremic cases. For ease of visualization, the horizontal axis is split into three sections: no circulating mf (n = 26, green dots), 1–50 mf/mL (n = 9, blue dots), and 51–20,000 mf/mL (n = 27, purple dots).

Of the 109 *L*. *loa* infected patients tested, 62 had known mf counts, including 26 amicrofilaremic patients. The mf levels of the 62 individuals with available microfilaremia data were plotted against the quantitative data reported by the LFA reader (Fig 5). When including all 62 data points, a 1-tail Spearman analysis revealed only a marginal correlation (r = 0.36, p = 0.0018) between test line intensity and microfilaremia. When restricting the analysis to the 36 people with circulating mf, the 1-tail Spearman analysis did not show a statistically significant correlation between the two variables (r = 0.16, p = 0.18). Of the 26 amicrofilaremic patients, 24 (92%) were positive in the LFA when read visually and had wide range of responses when quantified, from 0 to 1133 RUs (Fig 5).

## Discussion

This paper introduces the Loa Antibody Rapid Test developed by Drugs & Diagnostics for Tropical Diseases (San Diego, CA). The Loa Antibody Rapid Test adapts a recombinant *L*. *loa* antigen (*Ll*-SXP-1) already proven to be sensitive and specific in ELISA and LIPS assays [20,21] to a LFA platform, easily amenable to point-of-care use. An optional smartphone-based reader allows to quantify the test line intensity and to capture GPS coordinates, time stamp, and patient information on a secure server that is compliant with the Health Insurance Portability and Accountability Act (HIPAA). The Loa Antibody Rapid Test is a Research Use Only (RUO) device intended primarily for epidemiological purposes. It is not approved for *in vitro* diagnostic procedures or to support individual case management. Being an antibody based test, it does not discriminate previous from current infections. The test kit is commercially available and will remain so during the lifetime of the company. To our knowledge, the Loa Antibody Rapid Test is the first commercially available LFA device for the detection of *L*. *loa* infection. The test presented herein complements the immunoassays commercialized by others to detect serological responses to onchocerciasis and lymphatic filariasis [28]. These include antibody-based lateral flow assays specific for Ov16 (Alere, Waltham, USA [29]), Wb123 (Alere, [29]), Bm14 (Brugia Rapid; Reszon, Selangor, Malaysia, [30]) and Bm14+R1 (PanLF Rapid, Reszon [30]), and ELISA assays for Wb123 (InBios, Seattle, USA) and Bm14 (Cellabs, Brookvale, Australia [27]). Antigen-based tests remain much needed and currently exist only for lymphatic filariasis, where they detect circulating filarial antigen (CFA) on a rapid point-of-care platform (BinaxNOW Filariasis [31] and Filariasis Test Strip (FTS) [32], both by Alere) or by ELISA (TropBio Og4C3, Cellabs [33]). There is an emerging trend to use readers in combination with rapid tests. For instance, a densitometer was used to quantify the test results from the Filariasis Test Strip and estimate the *W*. *bancrofti* CFA levels in human blood, which are correlated with adult worm burdens. This technique may be useful for assessing the impact of treatment on adult *W*. *bancrofti* worms [34].

The Loa Antibody Rapid Test features novel reporter nanoparticles (gold nanoshells) and a bridged assay design, with *Ll*-SXP-1 being localized both at the surface of the nanoparticles and at the test line. A positive test occurs when an antibody specific for *Ll*-SXP-1 is present. The bivalent antibody can bridge between two antigens, one on the reporter nanoparticle and one at the test line, giving rise to a signal from the nanoparticle at the test line. The device has the capability to detect, at least in theory, any bivalent antibody specific for *Ll*-SXP-1, including IgGs and IgMs. In addition, there must be *two* simultaneous binding events to *Ll*-SXP-1 for the assay to be positive, which may have a favorable effect on the specificity. This design substantially differs from that of the published immunoassays for SXP-1 [20,21] and was selected because of promising initial results obtained with small data sets, not reported herein.

The assay develops rapidly and should be read after 20 minutes. The smartphone reader gives remarkably reproducible results, with coefficient of variations of 0.7–1.7% when measuring the same strip. Assay-to-assay reproducibility was also good, with CV values of 6.2–19% (Table 3). In addition, the assay demonstrated a strong analytical sensitivity and could detect antibody response in highly diluted serum samples (up to 800-fold dilutions).

The Loa Antibody Rapid Test was evaluated with 109 serum samples from patients with confirmed loiasis and additional samples from people with other filarial diseases and healthy controls. When read with the naked eye, the assay was 94% sensitive and 100% specific towards normal controls from endemic and non-endemic areas, and from people infected with *S*. *stercoralis*. There was some cross-reactivity with *O*. *volvulus*, *W*. *bancrofti*, and *M*. *perstans* infections, with specificity values of 82, 87, and 88% respectively. It is noteworthy that when read by eye, the performance of the Loa Antibody Rapid Test (94% sensitivity, 82–88% specificity) substantially exceeded that of the pan-IgG ELISA (67% sensitive, 81%, specific) and was closer to that observed for the fluorescence-based pan-IgG LIPS (94–100% sensitive, 78% specific).

Establishing a cutoff with the smartphone reader allowed us to tune the performance of the device. By selecting a cutoff of 600 RUs, the assay became 71% sensitive, 96% specific for *O*. *volvulus*, and 100% specific for *W*. *bancrofti* (Table 3). These values are comparable to the performance of the previously-developed IgG4-specific ELISA (47% sensitive, 99% specific, Supplementary Table 2). Using a cutoff of 600 RUs also allows to eliminate cross-reactivity with *M*. *perstans* (100% specificity), which is important since *M*. *perstans* can be the most abundant human filarial parasite co-endemic with *L*. *loa* [35].

Different applications will require different trade-offs between sensitivity and specificity. The end-user will be able to select the optimal compromise based on the Receiver Operating Characteristic (ROC) curves provided in Fig 4 and in the Supporting Information.

There was no correlation between test line intensity and microfilariae load. It should be stressed, however, that the device is neither intended nor approved for individual case management. The test is primarily devised for epidemiological studies and to support mapping projects for ivermectin-based MDA programs.

The prevalence of loiasis has been historically assessed in two different ways. The RAPLOA test is a questionnaire in which participants are asked for a history of eye worm. Alternatively, blood microfilariae can be counted with a standard or smartphone-based microscope. Because the microfilariae circulate with a diurnal periodicity, microscopic examinations must be carried out between 10 AM and 2–4 PM. The Loa Antibody Rapid Test offers a complementary way of determining the prevalence of loiasis, with the advantage that it can be used at any hour of the day, which should simplify the logistics of any mapping operation. Additionally, the Loa Antibody Rapid Test will detect past infections as well as active but amicrofilaremic cases. Therefore, the SXP-1 seroprevalence determined by the Loa Antibody Rapid Test could be substantially higher than the prevalence of *Loa* microfilariae determined by microscopy. If confirmed, this feature may be advantageous to map areas of low *Loa* endemicity while sampling only a reasonably small number of people per community. For the sake of comparison, the relationship between microfilaremia and seroprevalence has been established for *O*. *volvulus* in a recent survey of 35 Togolese villages, where the *O*. *volvulus* microfilaremia prevalence was 2.5% and the Ov16 seroprevalence was 19.7% [36].

A field evaluation is now warranted. Field tests should verify the sensitivity and specificity of the device with fresh blood samples. It should be noted that all specificity data were obtained with South American samples of *O*. *volvulus* and Asian samples of *W*. *bancrofti*. Geographical differences in the genetics of the organisms and in the host immune response may exist and their effect on the specificity of the assay should be assessed. Field tests should also focus on establishing correlations between microfilaremia and *Ll*-SXP-1 seroprevalence in different settings. Finally, the results of field studies should be used to refine the current models aimed at predicting the risk of SAEs [14]. It will be important to formally demonstrate if seroprevalence data can help increase the accuracy of the predictions.

In conclusion, the Loa Antibody Rapid Test is the first commercial test for loiasis, with an anticipated wholesale price in the $1–2 range. It is a sensitive and specific test adapted to map loiasis at the district and community levels. It is our hope that the maps will facilitate programmatic decisions in MDA campaigns against river blindness in zones that are co-endemic with *L*. *loa*. In addition, we hope that the Loa Antibody Rapid Test will eventually assist elimination campaigns directed at loiasis itself, a disease which affects over 10 million people and which deserves attention in its own right.

### Accession numbers

GenBank Accession Numbers: *B*. *malayi* antigen, partial (Bm14) AAA67319.1; *L*. *loa* SXP-1: EFO21235.1; *O*. *volvulus* Ov17: AAA18283.1.; *W*. *bancrofti* SXP antigen, partial: AAC70783.1.

## Supporting information

S1 Table *Loa loa* SXP-1 and its orthologs. Identity and similarity indexes were obtained by Protein BLAST (BLASTP).
(TIF)

S2 Table Historic data of the *Ll*-SXP-1 based ELISA and LIPS assays. Data from [21].
(TIF)

S1 Fig Comparison of the intensity of the control and test lines. A total of 446 assays were analyzed in this project and the control lines had a median value of 1321 RUs. The median value for the *Loa* samples was 845 RUs.
(TIF)

S2 Fig Receiver Operating Characteristic (ROC) curve. Adjusting the cutoff value above which a test is deemed positive allows to modulate its sensitivity and specificity. The graph shows the sensitivity that can be expected of the Loa Antibody Rapid Test for a given specificity versus *O. volvulus* infection.
(TIF)
