# Supplementary figures and images for: A novel rapid test for detecting antibody responses to Loa loa infections
Source: PLoS Negl Trop Dis. 2017 Jul 27;11(7):e0005741. doi: 10.1371/journal.pntd.0005741 (PMC5531435; doi:10.1371/journal.pntd.0005741)

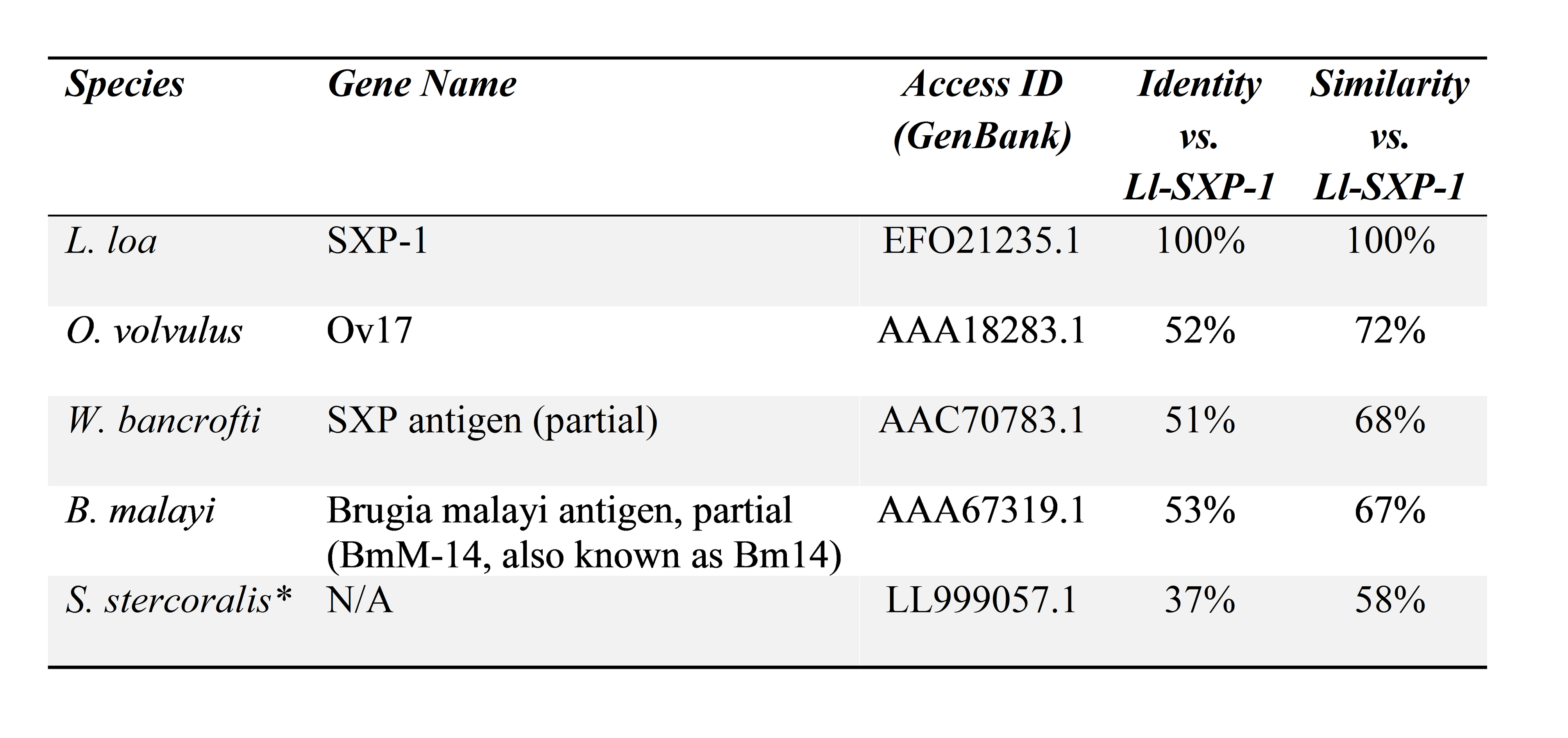

Supplement: S1 Table — Identity and similarity indexes were obtained by Protein BLAST (BLASTP). (TIF) [file pntd.0005741.s001.tif]

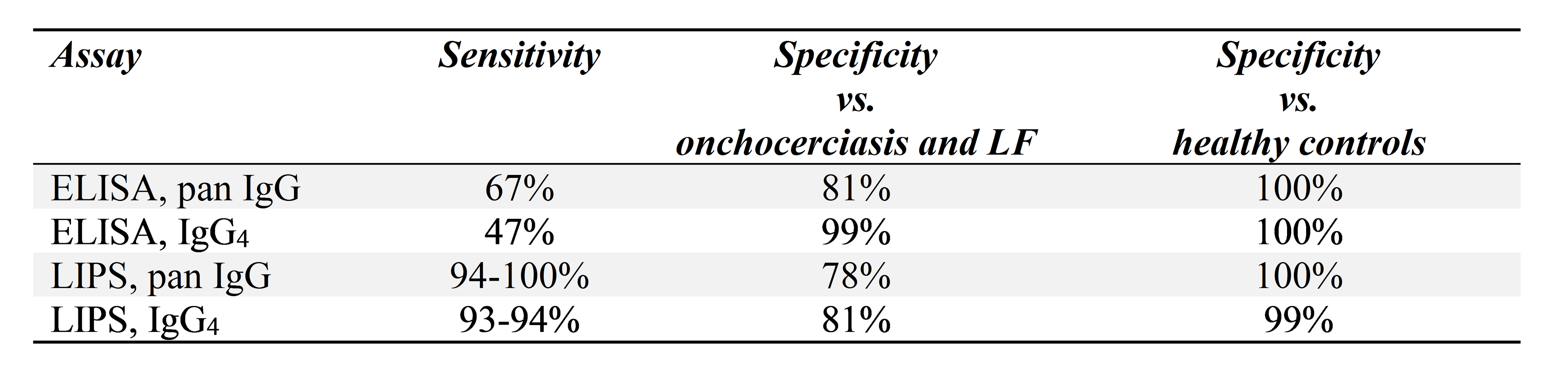

Supplement: S2 Table — Data from [21]. (TIF) [file pntd.0005741.s002.tif]

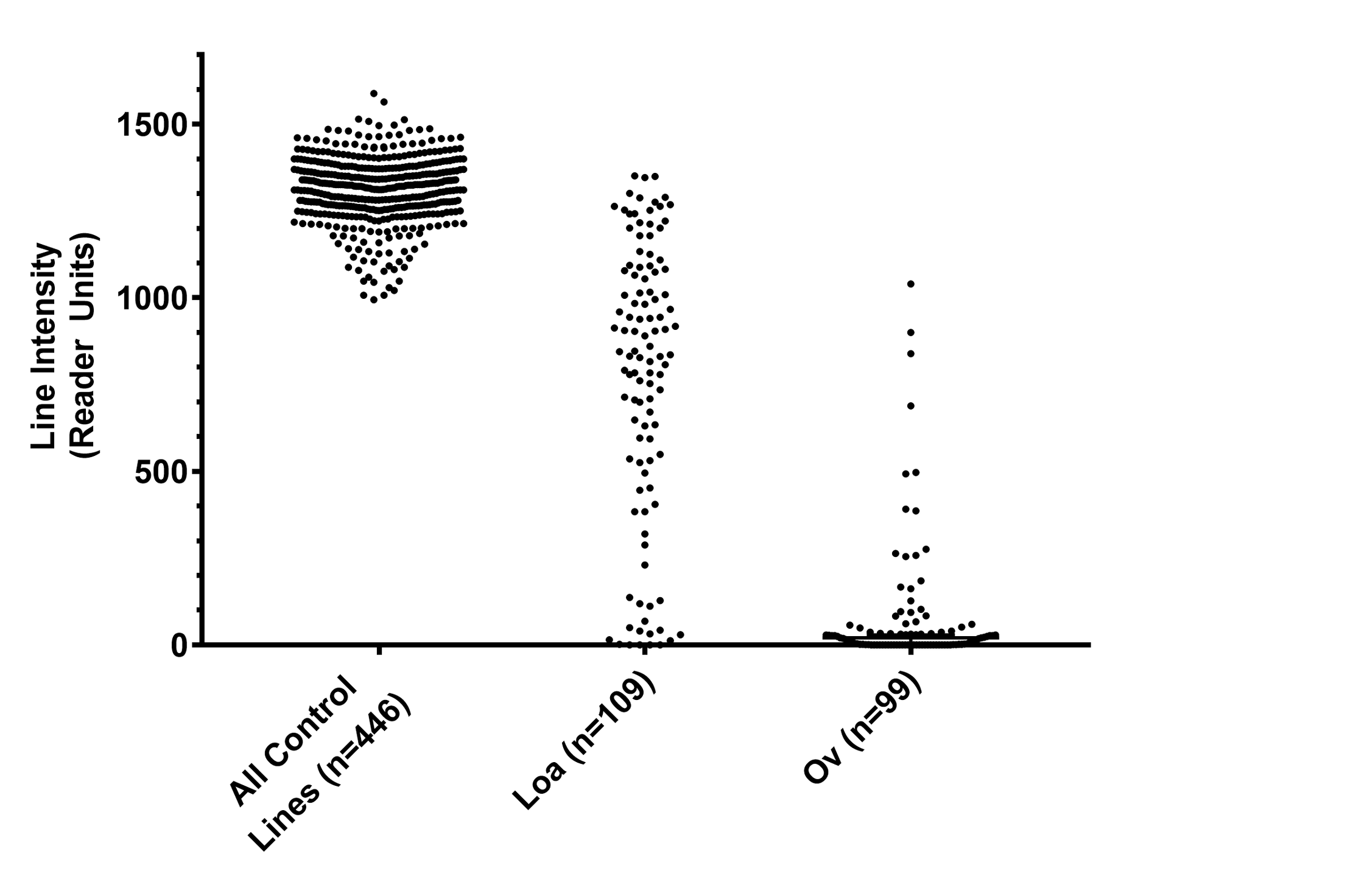

Supplement: S1 Fig — A total of 446 assays were analyzed in this project and the control lines had a median value of 1321 RUs. The median value for the Loa samples was 845 RUs. (TIF) [file pntd.0005741.s003.tif]

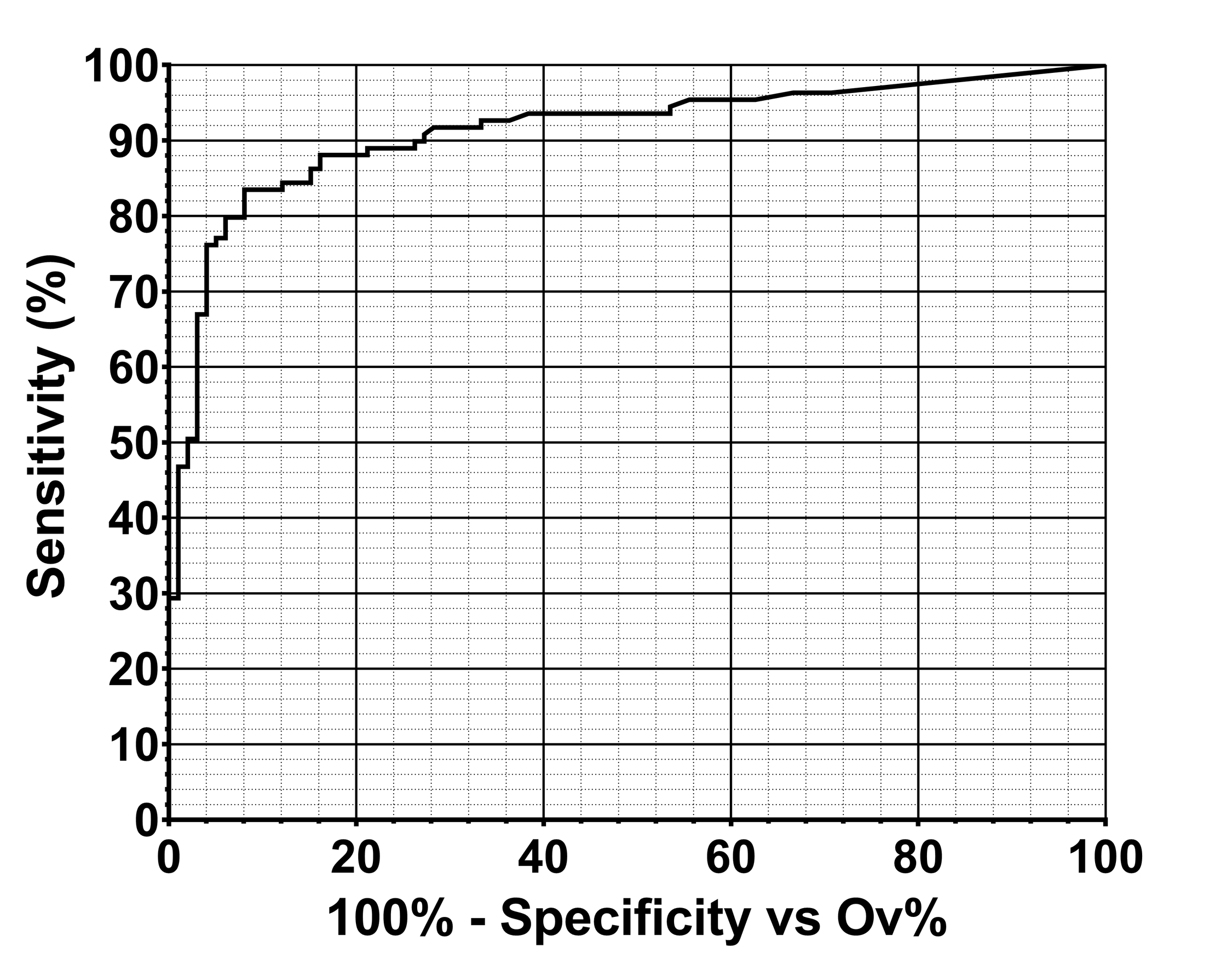

Supplement: S2 Fig — Adjusting the cutoff value above which a test is deemed positive allows to modulate its sensitivity and specificity. The graph shows the sensitivity that can be expected of the Loa Antibody Rapid Test for a given specificity versus O. volvulus infection. (TIF) [file pntd.0005741.s004.tif]
